# Supplementary figures and images for: YES1 as a Therapeutic Target for HER2-Positive Breast Cancer after Trastuzumab and Trastuzumab-Emtansine (T-DM1) Resistance Development
Source: Int J Mol Sci. 2021 Nov 26;22(23):12809. doi: 10.3390/ijms222312809 (PMC8657782; doi:10.3390/ijms222312809)

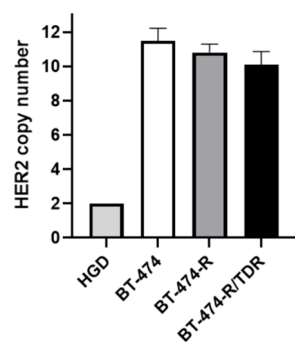

Figure S1

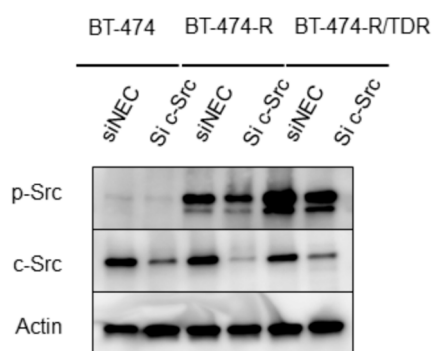

(A)

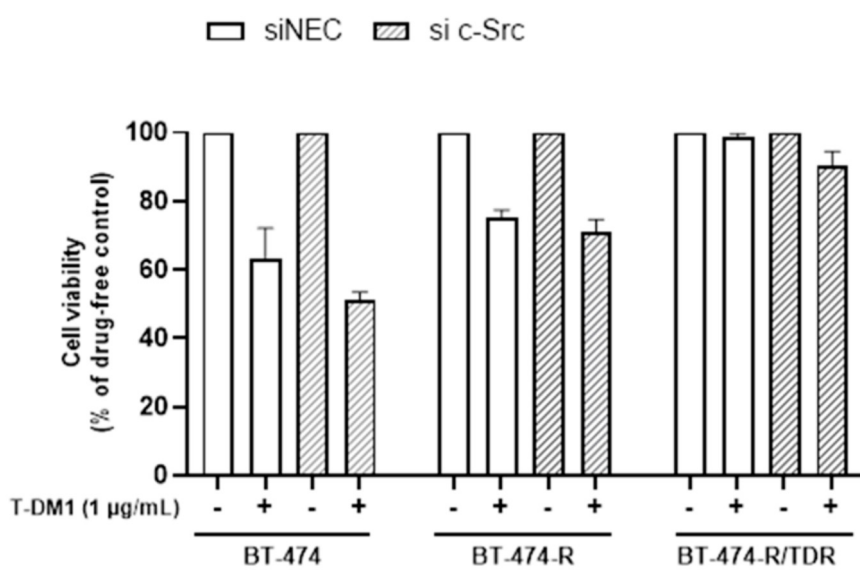

(B)

Figure S2

Supplement: Supplementary file 1 [file ijms-22-12809-s001.zip › ijms-1423399-supplementary.pdf]
